# An Improved BM3D Algorithm Based on Image Depth Feature Map and Structural Similarity Block-Matching

**DOI:** 10.3390/s23167265

**Published:** 2023-08-18

**Authors:** Jia Cao, Zhenping Qiang, Hong Lin, Libo He, Fei Dai

**Affiliations:** 1 College of Big Data and Intelligent Engineering, Southwest Forestry University, Kunming 650224, China; caojia@swfu.edu.cn (J.C.); linh1226@swfu.edu.cn (H.L.); daifei@swfu.edu.cn (F.D.); 2 Information Security College, Yunnan Police College, Kunming 650221, China; helibo2015@163.com

**Keywords:** image denoising, BM3D, block-matching, feature map, *SSIM*

## Abstract

We propose an improved BM3D algorithm for block-matching based on UNet denoising network feature maps and structural similarity (*SSIM*). In response to the traditional BM3D algorithm that directly performs block-matching on a noisy image, without considering the deep-level features of the image, we propose a method that performs block-matching on the feature maps of the noisy image. In this method, we perform block-matching on multiple depth feature maps of a noisy image, and then determine the positions of the corresponding similar blocks in the noisy image based on the block-matching results, to obtain the set of similar blocks that take into account the deep-level features of the noisy image. In addition, we improve the similarity measure criterion for block-matching based on the Structural Similarity Index, which takes into account the pixel-by-pixel value differences in the image blocks while fully considering the structure, brightness, and contrast information of the image blocks. To verify the effectiveness of the proposed method, we conduct extensive comparative experiments. The experimental results demonstrate that the proposed method not only effectively enhances the denoising performance of the image, but also preserves the detailed features of the image and improves the visual quality of the denoised image.

## 1. Introduction

In the process of image generation and transformation, noise is inevitably generated, which not only reduces the visual quality of the image but also affects subsequent advanced visual processing tasks [1]. Therefore, it is necessary to take measures to eliminate noise while preserving image details.

At present, image-processing algorithms can be roughly divided into three categories: spatial-domain denoising, frequency-domain denoising [2,3], and deep-learning denoising. Spatial-domain denoising involves directly averaging pixels on noisy images, such as Gaussian filtering [4], median filtering [5], and anisotropic filtering [6]. This type of method can effectively preserve the edge information of the image, but it is also prone to blurring the image. Frequency-domain denoising is the process of converting an image from the spatial domain to the transform domain. In the transform domain, the image is divided into two parts: low-frequency and high-frequency, and noise is more present in the high-frequency components of the image. Therefore, the purpose of denoising can be achieved by processing the high-frequency components through threshold methods [7]. In 2005, based on the nonlocal self-similarity of images, Buades, Coll, and Morel [8] proposed a spatial-domain denoising algorithm called NLM, which improved the performance of the spatial-domain denoising algorithm to a new height. To further improve the performance of the denoising algorithm, in 2007, Dabov, Foi et al. [9] proposed the BLOCK-MATCHING and 3D filtering (BM3D) algorithm that has obvious denoising effects and can effectively retain image details, combining the characteristics of nonlocal self-similarity and frequency-domain denoising. The BM3D algorithm can be simply summarized into three steps [10]: the first step is block-matching grouping, which involves dividing the noisy image into blocks. Based on certain similarity measurement criteria and set threshold, search for similar blocks of each block and combine them to form a 3D matrix cluster; The second step is to use a filtering method to filter each cluster; The third step is aggregation, which involves aggregating the filtered clusters to obtain the output image. Ref. [11] proposed quantum mechanics-based signal and image representation (QAB), which processes high and low values in signals or images by thresholding coefficients. This method has interesting potential in denoising signals and images and is very suitable for Poisson and speckle noise. Inspired by quantum multibody theory, ref. [12] proposed an image-denoising algorithm called De-QuIP. Based on patch analysis, a term similar to quantum mechanics is used to formalize the similarity measure in the local image neighborhood, effectively preserving the local structure of the real image. With the development of artificial intelligence technology, deep-learning-based denoising methods have attracted increased attention. Ref. [13] introduced a nonlocal self-similarity before network processing flow and proposed a blind-spot-based self-supervised image-denoising method. This method can significantly improve denoising performance. Ref. [14] proposed a joint patch-group-based sparse representation (JPG-SR), which can effectively integrate the local sparsity and nonlocal self-similarity of images. The objective and perceptual quality obtained by applying this method to image restoration problems is superior to many state-of-the-art methods. Ref. [15] proposed a group sparsity residual constraint with nonlocal priors (GSRC-NLP) for image restoration to address the problem of traditional GSR models being unable to faithfully estimate image sparsity. This algorithm has achieved excellent results in image denoising, image restoration, and other aspects. Ref. [16] proposed a convolutional neural network called BM3D-Net, which unrolls the BM3D algorithm into a convolutional neural network structure, and “extraction” and “aggregation” layers are utilized to model the block-matching process of traditional BM3D. The denoising effect achieved is significantly better than that of the BM3D method. Ref. [17] proposed a deep neural network called DIVA based on the theory of quantum many-body physics and designed a baseline adaptive denoising algorithm (De-QuIP). This algorithm can handle nonlocal image structures, significantly reducing training costs while improving the visual quality of denoising. Ref. [18] proposed a deep denoising network embedded in quantum interactions, incorporating nonlocal structures into the proposed CNN architecture. The key feature integration in quantum theory enables this method to effectively handle image-denoising problems. Ref. [19] proposed a nonlocal denoising convolutional neural network (NL-DeCNN) network for denoising vibration signals of rolling bearings, and nonlocal mean (NLM) was introduced to construct nonlocal blocks (NLB), which improved the denoising performance of CNN.

According to the BM3D step, block-matching is a critical procedure of the algorithm. Currently, there has been a lot of research on block-matching in the academic community. Refs. [20,21,22] have proposed a new block similarity measure to alleviate the interference of noise and ensure the accuracy of block-matching in images with high-intensity noise. Refs. [23,24] proposed an improved BM3D technology based on an adaptive threshold, which has excellent denoising performance for different types of noise in different images. In [25], to achieve more accurate block-matching, each image is divided into three different regions using the region energy of the alternating current (AC) coefficients of discrete cosine transform (DCT), and the block size is adaptively selected based on the region. It can be seen that the current block-matching improvement algorithms have achieved certain results from the perspective of improving similarity measurement criteria and improving block-matching accuracy. However, the block-matching operations involved in the above studies often directly process noisy images, lacking consideration for deep-level features of the image, such as structure, brightness, contrast, etc. This leads to the neglect of certain important features of the image, resulting in inaccurate block-matching, as a result, the visual quality of the denoised image may not necessarily be good.

In recent years, the rapid development of deep learning has provided ideas for the extraction of deep features in images. Deep learning has the advantages of autonomous learning of target features and a high recognition rate [26,27]. The RGB-D saliency object detection model established in [28] utilized ResNet-50 [29] and VGG-16 [30] to process RGB images and their corresponding depth maps, which can effectively consider the complementarity of multi-level features and generate high-quality predictive saliency maps. Ref. [31] proposed the fusion of spatial and temporal feature representations of speech emotion by parallelizing a Transformer encoder for speech emotion recognition and convolutional neural networks (CNNs). This method not only significantly improved the results of emotion recognition but also reduced computing costs. Ref. [32] proposed a multi-attention-based UNet (MA-UNet) [33] to improve the extraction ability of fine-grained features, which was applied to the semantic segmentation of remote sensing images and achieved good results. Ref. [34] and others utilized feature fusion technology to construct a pyramid structure feature map with equally rich positional and semantic information, and performed object detection on multi-scale feature layers, enhancing the network’s adaptability to scale images. It can be seen that deep learning can learn the deep-level features of images, and using these features to process other advanced visual tasks can achieve more accurate and effective results. However, there is currently little research on using deep-learning feature maps for block-matching. Therefore, inspired by the above research, we adopt a pre-trained UNet denoising network [35] to extract feature maps of different scales from noisy images. Unlike the traditional BM3D algorithm for block-matching on the noisy image, the improved BM3D algorithm proposed in this paper first performs block-matching on the feature maps. Based on the results of block-matching on each feature map, all corresponding similar blocks are searched for at the same location in the noisy image, thus obtaining a set of block-matching that considers different scale features of the noisy image.

In addition, the similarity measurement for block-matching in the traditional BM3D algorithm is based on pixels to calculate the similarity between two images, which is too simple and direct to consider the depth features of the noisy image. The *SSIM* [36] is an index utilized to measure the similarity between two images. It fully considers the structure, brightness, and contrast information of the image itself, which is more in line with human visual perception. Therefore, to enhance the visual quality of denoised images, we utilize *SSIM* to improve the traditional similarity measurement and propose a new similarity measurement criterion that considers the structural similarity of noisy images. The contributions of this paper are three-fold:

(1) A pre-trained UNet denoising network is utilized to obtain feature maps at different scales of the noisy image, and then block-matching operations of the BM3D algorithm are performed on the feature maps. The block-matching results are mapped to the corresponding positions of the noisy image, therefore obtaining block-matching groups that take into account the depth characteristics of the noisy image.

(2) Considering that *SSIM* has the characteristics of image structure, brightness, and contrast, an improved similarity measurement criterion based on *SSIM* is proposed.

(3) Based on classic image data, extensive comparative experiments and depth analysis are conducted to explain the value of considering image depth features for block-matching operations proposed in this paper.

The other parts of this paper are organized as follows. The Section 2 reviews the basic principles of the traditional BM3D algorithm, while the Section 3 introduces the methods proposed in this paper, including the modified block-matching based on noisy image depth features and the improved similarity measurement based on *SSIM*. The Section 4 conducts extensive experiments to verify the effectiveness of the proposed improvement method. Section 5 offers some conclusions.

## 2. Traditional BM3D Algorithm

**Figure 1:**
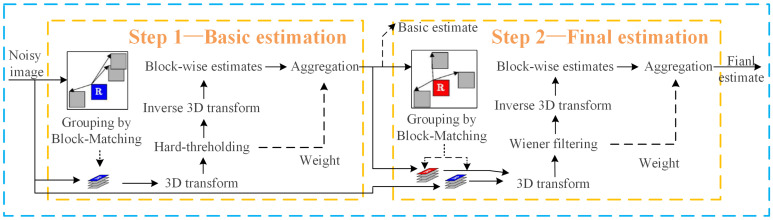
Flow chart of the traditional BM3D algorithm.

The BM3D algorithm is mainly divided into two steps. The first step is the basic estimation, and the second step is the final estimation. The first and second steps both include block-matching grouping, filtering, and aggregation. The execution process of the algorithm is shown in Figure 1.

### 2.1. Basic Estimation

#### 2.1.1. Block-Matching Grouping

Construct a two-dimensional sliding window with a size of $N_{1}\times N_{1}$, and take image blocks based on the set step size *p* on the noisy image *I*. Set the current block being operated on as $Z_{R}$. Then search for similar blocks of the reference block $Z_{R}$ in the $N_{2}\times N_{2}$ search area centered around $Z_{R}$, denoted as $Z_{Q}$. Define the distance $d\left(Z_{R},Z_{Q}\right)$ (inversely proportional to similarity) to measure the similarity between $Z_{R}$ and $Z_{Q}$, and its expression is shown in Equation (1).

(1) $$d\left(Z_{R},Z_{Q}\right)=N_{1}^{-1}{∥\gamma \left(\tau_{2D}\left(Z_{R}\right),\lambda_{\mathrm{thr}2D}\sigma \sqrt{2\log\left(N_{1}^{2}\right)}\right)-\gamma \left(\tau_{2D}\left(Z_{Q}\right),\lambda_{\mathrm{thr}2D}\sigma \sqrt{2\log\left(N_{1}^{2}\right)}\right)∥}_{2}$$

where $\tau_{2D}$ is the 2D unitary transformation operator (such as DCT, DFT, etc.). γ is a hard threshold operator, $\lambda_{\mathrm{thr}2D}$ is a fixed threshold parameter, σ represents the standard deviation of noise, ${∥•∥}_{2}$ represents a binomial. The definition of γ is as follows.

(2) $$\gamma \left(\lambda ,\lambda_{\mathrm{thr}}\right)=\left\{\begin{array}{cc} \lambda & |\lambda |>\lambda_{\mathrm{thr}}\\ 0 & \mathrm{otherwise} \end{array}\right.$$

**Figure 2:**
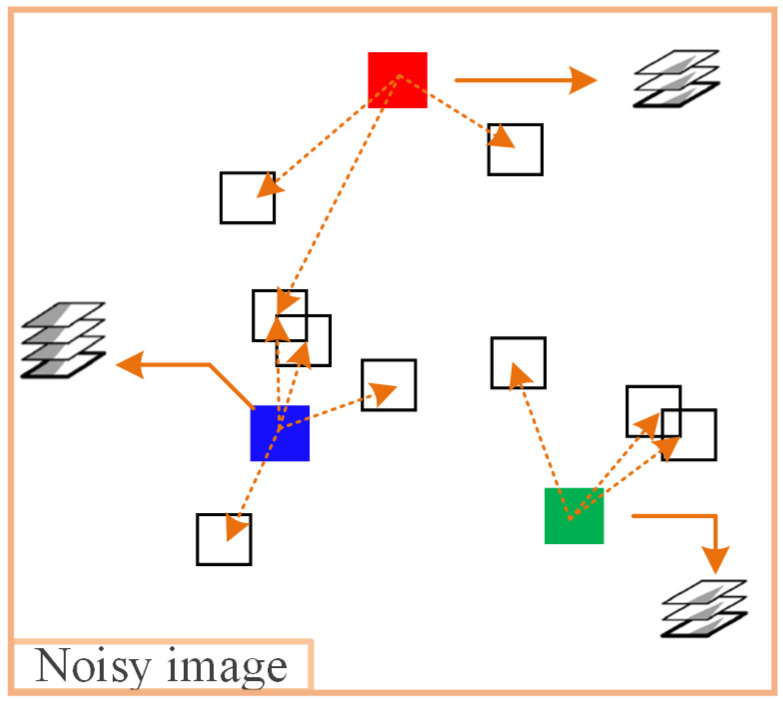
A simple example of block-matching grouping (in the figure, three reference blocks are filled with red, green, and blue colors, while the rest are similar blocks that are matched to each other. The dotted arrows point to the similar blocks to be matched, while the solid arrows point to the 3D similar blocks matrix.).

The idea of finding similar blocks for reference block $Z_{R}$ is to select an appropriate threshold $\tau_{1}$, and if $d\left(Z_{R},Z_{Q}\right)<\tau_{1}$, $Z_{Q}$ is considered similar to $Z_{R}$ and added to the set $B_{R}$. Finally, integrate the reference block $Z_{R}$ and their similar block sets into a three-dimensional matrix $T_{R}$. Figure 2 is a simple example of block-matching grouping on the noisy image.

#### 2.1.2. Hard Threshold Shrinkage

Perform a three-dimensional unitary transformation $\tau_{3D}^{\mathrm{ht}}$ (two-dimensional Bior wavelet transform and one-dimensional Haar wavelet transform) on a three-dimensional matrix $T_{R}$. Three-dimensional transform domain denoising can separate the noise and real information of an image while ensuring no energy loss. Most of the real information of the image is concentrated at the vertex of the 3D matrix energy, while the noise is often concentrated at the bottom of the 3D matrix energy. Therefore, through hard threshold filtering in the transformation domain, most of the image information can be preserved while effectively reducing the noise. $V_{{}_{R}}^{\mathrm{ht}}=\tau_{{}_{3D}}^{-1\mathrm{ht}}\left(\gamma \left(\tau_{{}_{3D}}^{\mathrm{ht}}\left(T_{{}_{R}}\right),\lambda_{\mathrm{thr}3D}\sigma \sqrt{2\log(N_{1}^{2})}\right)\right)$ is the set of estimated values for blocks in $B_{R}$ after hard threshold filtering, and $\lambda_{\mathrm{thr}3D}$ is a fixed threshold parameter.

#### 2.1.3. Aggregation

After hard threshold shrinkage, each block will obtain a basic estimated weight value, $N_{R}$ represents the number of non-zero coefficients in the matrix $V_{{}_{R}}^{\mathrm{ht}}$. The basic estimated weight value $\omega_{R}^{\mathrm{basic}}$ of the reference block is shown in Equation (3).

(3) $$\omega_{R}^{\mathrm{basic}}=\left\{\begin{array}{cc} \frac{1}{N_{R}} & N_{R}\geq 1\\ 1 & N_{R}=0 \end{array}\right.$$

For a pixel *i* that may appear in multiple blocks, it is necessary to perform a weighted average on the estimated values of these overlapping blocks to obtain the basic estimation value of *i*. The formula is as follows:

(4) $$Y_{\mathrm{basic}}\left(i\right)=\frac{\sum_{B_{R}}\sum_{Z_{Q}\in B_{R}}\omega_{R}^{\mathrm{basic}}\cdot V_{RQ}^{\mathrm{ht}}}{\sum_{B_{R}}\sum_{Z_{Q}\in B_{R}}\omega_{R}^{\mathrm{basic}}\cdot x_{Q}},\forall i\in I$$

where $Z_{Q}$ represents any image block containing pixel *i* in the set $B_{R}$, and similarly, there may be multiple sets $B_{R}$ with image blocks containing pixel *i*. Therefore, it is necessary to weighted-average the estimated values of all blocks containing that pixel in the set. $V_{{}_{RQ}}^{\mathrm{ht}}$ is the estimated value of the block $Z_{Q}$ containing pixel *i* in any set $B_{R}$, $x_{Q}$ is a 0–1 variable, and the expressions for $V_{{}_{RQ}}^{\mathrm{ht}}$ and $x_{Q}$ are as follows:

(5) $$V_{RQ}^{\mathrm{ht}}=\left\{\begin{array}{cc} V_{RQ}^{\mathrm{ht}} & i\in Z_{Q}\\ 0 & i\notin Z_{Q} \end{array}\right.$$

(6) $$x_{Q}=\left\{\begin{array}{cc} 1 & i\in Z_{Q}\\ 0 & i\notin Z_{Q} \end{array}\right.$$

### 2.2. Final Estimation

#### 2.2.1. Block-Matching Grouping

Based on the basic estimation image $Y_{\mathrm{basic}}$ obtained in the first step, block-matching grouping is performed again to form a new three-dimensional matrix $T_{R2}$. There are two three-dimensional matrices at this time, one is the three-dimensional matrix $T_{R}$ composed of similar blocks in the noisy image obtained in the first step, and the other is the three-dimensional matrix $T_{R2}$ composed of similar blocks in the image generated from the basic estimation.

#### 2.2.2. Collaborative Wiener Filtering

Unlike the use of hard threshold filtering in the basic estimation stage, the final estimation utilizes Wiener filtering. The attenuation co-efficient of the Wiener filter is calculated as follows:

(7) $$W_{R}^{\mathrm{final}}=\frac{{\left|\tau_{3D}^{\mathrm{wie}}\left(T_{R2}\right)\right|}^{2}}{{\left|\tau_{3D}^{\mathrm{wie}}\left(T_{R2}\right)\right|}^{2}+\sigma^{2}}$$

Then, the Wiener filtering of $T_{R}$ is achieved by multiplying the 3D transform co-efficient $\tau_{3D}^{\mathrm{wie}}\left(T_{R}\right)$ of the noisy image with the Wiener contraction co-efficient. Finally, the estimation value of the block is generated through inverse transformation. The expression for Wiener filtering is as follows:

(8) $$V_{R}^{\mathrm{wie}}=\tau_{3D}^{-1\mathrm{wie}}\left(W_{R}^{\mathrm{final}}\tau_{3D}^{\mathrm{wie}}\left(T_{R}\right)\right)$$

The weight of each reference block in the final estimation stage can be defined as:

(9) $$\omega_{R}^{\mathrm{final}}=\sigma^{-2}{∥W_{R}^{\mathrm{final}}∥}_{2}^{-2}$$

#### 2.2.3. Aggregation

Similarly, it is necessary to perform a weighted average on these block estimates to obtain the final estimate of the pixel *i*. The formula is as follows:

(10) $$Y_{\mathrm{final}}\left(i\right)=\frac{\sum_{B_{R}}\sum_{Z_{Q}\in B_{R}}\omega_{R}^{\mathrm{final}}\cdot V_{RQ}^{\mathrm{wie}}}{\sum_{B_{R}}\sum_{Z_{Q}\in B_{R}}\omega_{R}^{\mathrm{final}}\cdot x_{Q}},\forall i\in I$$

where $V_{RQ}^{\mathrm{wie}}$ is the estimated value of the block $Z_{Q}$ containing pixel *i* in any set $B_{R}$, and its expression is similar to Equation (5).

## 3. Proposed Method

The process of searching for similar blocks in the traditional BM3D algorithm is carried out on the noisy image, which does not take into account the deep-level features of the noisy image and may lead to inaccurate block-matching results. To fully consider the depth features at different scales of the noisy image and improve the accuracy of block-matching, we propose the modified block-matching based on noisy image depth features and the improved similarity measurement based on *SSIM*. The specific instructions are as follows.

### 3.1. The Modified Block-Matching Based on UNet Denoising Network Feature Maps

**Figure 3:**
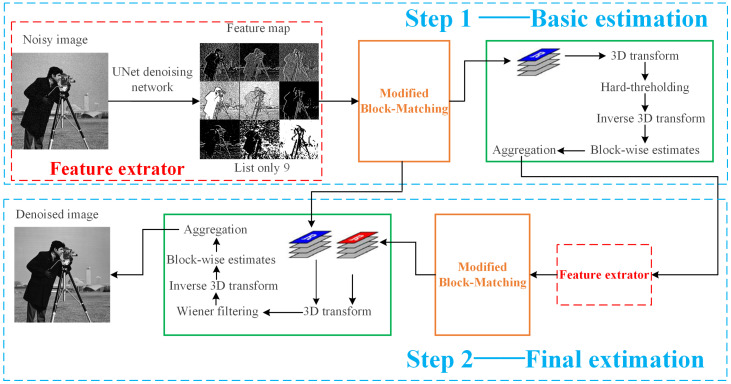
The improved BM3D algorithm flowchart based on UNet network feature maps.

Given the advantages of deep-learning networks in extracting deep-level features of images, we select a pre-trained UNet denoising network to extract feature maps of the noisy image, and then search for similar blocks on the feature maps. Based on the results of block-matching on each feature map, all corresponding similar blocks are searched for at the same position in the noisy image, thus obtaining a set of similar blocks that consider the deep-level features of the noisy image. Based on this, subsequent operations on BM3D are carried out. The execution process of the improved BM3D algorithm is shown in Figure 3.

**Figure 4:**
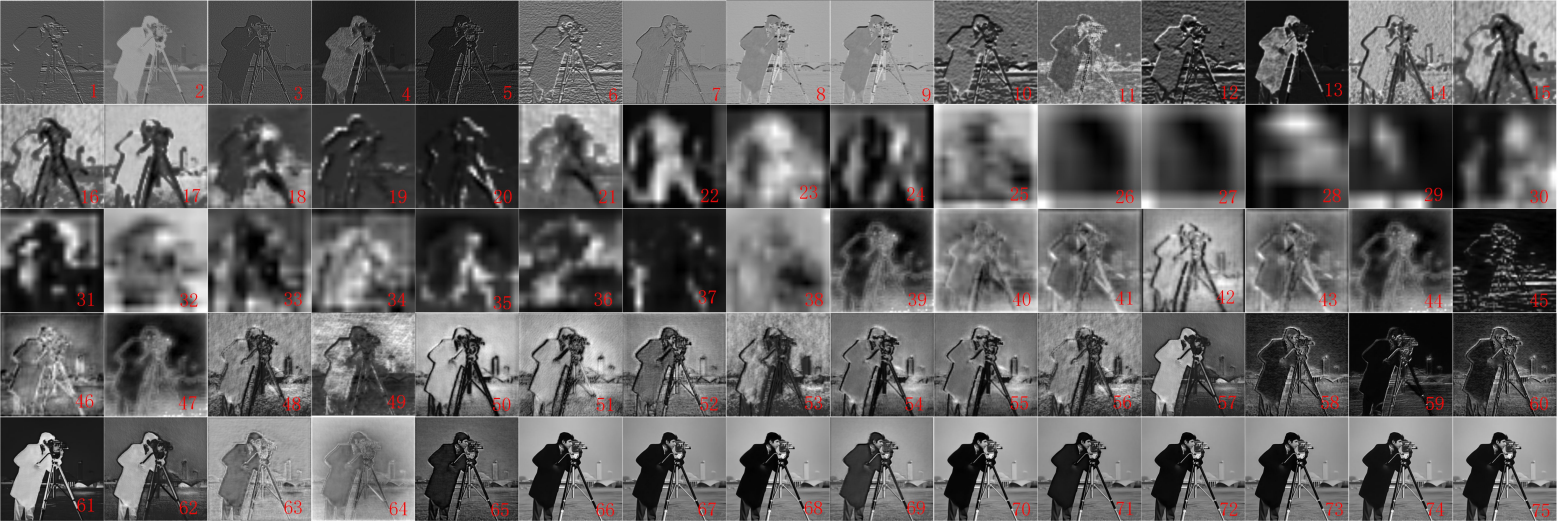
Feature maps of each layer of UNet network (where 1 to 75 respectively represent the serial numbers of the feature maps of each layer of the UNet network).

**Figure 5:**
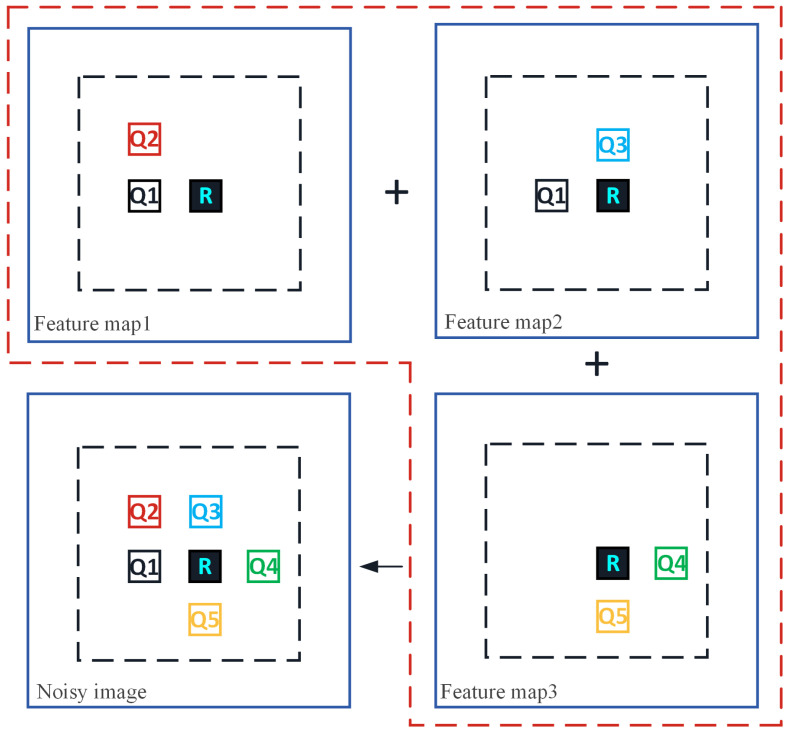
A simple example of the modified block-matching (where the dotted box represents similar block-matching on the feature map).

Compared with the flowchart of the traditional BM3D algorithm in Figure 1, it can be seen that the improved BM3D algorithm in this paper adds a feature extractor module before the block-matching of the traditional BM3D algorithm, which first inputs the noisy image into a pre-trained UNet denoising network to obtain feature maps at different scales of the noisy image. Figure 3 only lists 9 feature maps of different layers. The UNet denoising network consists of 4 processes, with process 1 being the head and having 9 layers with 48 channels per layer. Process 2 is down, with 20 layers and 48 channels per layer. Process 3 is up, with 36 layers and 96 channels per layer. Process 4 is last, with 10 layers and 3 channels per layer. Select a feature map from all channels in each layer and arrange it to form the feature maps of each layer of the UNet network shown in Figure 4. It can be seen that the UNet network has many layers, and the number of channels varies greatly among different process layers. The head and down processes of UNet networks are similar to the encoder structure and can compress images. The up and last processes are similar to the decoder structure, which can restore the image to the denoised image, making the image features more obvious. The last layer obtains the denoised image of the noisy image. Therefore, to fully consider the deep-level features of the noisy image, improve the accuracy of block-matching, and enhance the denoising effect of the BM3D algorithm, we select the feature maps of layers in the last process for improved block-matching. Figure 5 is a simple example of the modified block-matching module.

Unlike Figure 2, which performs a similar block search on the noisy image, as shown in Figure 5, it is assumed that three feature maps of the noisy image are selected from the UNet network, namely feature map 1, feature map 2, and feature map 3. Then search for similar blocks of reference block R on three feature maps, where the similar blocks of R in feature map 1 are block Q1 and block Q2, the similar blocks of R in feature map 2 are block Q1 and block Q3, and the similar blocks of R in feature map 3 are block Q4 and block Q5. Then, based on the matching of similar block positions on each feature map, select all non-repetitive similar blocks at the same positions on the noisy image, and use them as a set of similar blocks for reference block R. The set of similar blocks selected through the above process fully considers the impact of different scale features of the noisy image on block-matching, improving the accuracy of block-matching.

The next step is to input the 3D matrix formed by the set of similar blocks of each block into the next step, which is the same process as the traditional BM3D algorithm. Similarly, after the first step of algorithm execution, the basic estimated image needs to undergo feature map extraction and modified block-matching. Similar block sets that consider deep-level features are obtained from the basic estimated image and merged to form a 3D matrix $T_{R2}$. Then, the 3D matrix $T_{R}$ obtained from the first step on the noisy image and $T_{R2}$ are inputted into the next step for subsequent operations.

### 3.2. The Improved Similarity Measurement Criteria Based on SSIM

From Equation (1), it can be seen that the similarity measurement in the traditional BM3D algorithm is a simple operation based on the pixels of the image, which fails to consider the structural features of the image itself. *SSIM* is a measure of image similarity, which effectively integrates the structure, brightness, and contrast information of the image. These features can promote human visual perception and have the advantage of considering image structural features. The expression of *SSIM* is shown in Equation (11). Therefore, to further enhance the correlation between the BM3D algorithm and the structural features of the image itself, and improve the visual quality of the denoised image, we utilize *SSIM* to improve Equation (1). The improved similarity measurement criteria are shown in Equation (12):

(11) $$SSIM\left(Z_{R},Z_{Q}\right)=\frac{\left(2\mu_{R}\mu_{Q}+C_{1}\right)\left(2\sigma_{RQ}+C_{2}\right)}{\left(\mu_{R}^{2}+\mu_{Q}^{2}+C_{1}\right)\left(\sigma_{R}^{2}+\sigma_{Q}^{2}+C_{2}\right)}$$

where $\mu_{R}$ is the average of the reference block $Z_{R}$, $\mu_{Q}$ is the average of the block $Z_{Q}$, $\sigma_{R}^{2}$ is the variance of the reference block $Z_{R}$, $\sigma_{R}^{2}$ is the variance of the block $Z_{Q}$, and $\sigma_{RQ}$ is the covariance between $Z_{R}$ and $Z_{Q}$. $C_{1}$ and $C_{2}$ are constants that maintain stability, where $C_{1}=0.0004$, $C_{2}=0.0036$ in the experiment.

(12) $$d_{\mathrm{modified}}\left(Z_{R},Z_{Q}\right)=d\left(Z_{R},Z_{Q}\right)\times \left[1-SSIM\left(Z_{R},Z_{Q}\right)\right]$$

In Equation (12), the smaller the distance $d(Z_{R},Z_{Q})$, the closer the distance between the reference block $Z_{R}$ and the block $Z_{Q}$, indicating a higher similarity between the two blocks. $SSIM(Z_{R},Z_{Q})$ is a number between 0 and 1, and the larger it is, the higher the structural similarity between $Z_{R}$ and $Z_{Q}$, and the smaller $1-SSIM(Z_{R},Z_{Q})$. According to Equation (12), the distance $d_{\mathrm{modified}}\left(Z_{R},Z_{Q}\right)$ between $Z_{R}$ and $Z_{Q}$ is also smaller. In summary, the smaller $d_{\mathrm{modified}}\left(Z_{R},Z_{Q}\right)$, the higher the similarity between $Z_{R}$ and $Z_{Q}$. It can be seen that the improved similarity measurement formula $d_{\mathrm{modified}}\left(Z_{R},Z_{Q}\right)$ not only measures the pixel value similarity between blocks but also takes into account the inherent features such as the structure between blocks due to the introduction of the *SSIM*. This has a good promoting effect on improving the visual quality of the denoised image.

## 4. Experimental Results and Analysis

To verify the denoising performance of the improved BM3D method proposed in this paper, we conduct extensive comparative experiments, and the experimental results are presented and discussed. First, based on the combination of different layer feature maps using the UNet denoising network to improve the denoising effect of BM3D, the optimal feature map combination is selected. Second, compare the improved BM3D method proposed in this paper with other classic denoising algorithms and display the denoising effect diagram. Then, compare the denoising performance of various denoising algorithms on images with different noise levels. Finally, the contributions of different components in the proposed method are analyzed.

### 4.1. Data and Parameter Settings

In this experiment, we utilize classic test images for image denoising, such as Cameraman, boat, and Baboon. Each image is a Grayscale with a size of 256 × 256, including both noiseless images and images with various noise levels. We adopt pre-trained UNet network models on datasets with different noise levels and utilize these UNet models to extract feature maps of images with different noise levels for experiments. In addition, we utilize the peak signal-to-noise ratio (*PSNR*) and *SSIM* as evaluation indicators for image-denoising performance. The larger the *PSNR*, the better the denoising effect. The larger the *SSIM*, the higher the visual quality of the denoised image. The parameter settings for traditional BM3D and improved BM3D algorithm are the same, as shown in Table 1.

### 4.2. Selection of the Optimal Combination of Feature Maps

The UNet network has four processes: head, down, up, and last. Since feature maps of each layer in each process are similar, we select the last layer for each process to conduct experiments. A total of four layers are selected, namely head.1.block.1 (feature layer 1), down_Path.4.block.1 (feature layer 2), up_Path.4.conv_2.Block.1 (feature layer 3) and last.2 (feature layer 4). Due to the fact that each layer has multiple channels, there are many combinations of feature maps, including feature map combinations of different channels in any of the above four layers. The main purpose of this paper is to verify that the denoising performance of the improved BM3D algorithm based on deep feature maps is superior to the traditional BM3D algorithm. Therefore, for the convenience of the experiment, the way of obtaining feature map combinations in this experiment is as follows: first, select any two layers from the above four layers, with a total of 10 combinations, including 1&1 (representing feature layer 1 and feature layer 1), 1&2 (representing feature layer 1 and feature layer 2, and so on), 1&3, 1&4, 2&2, 2&3, 2&4, 3&3, 3&4, and 4&4. Then select one channel from each of these two layers and treat the selected two channels as a combination, without considering other combinations. Based on the principle of feature map combination selection above, we calculate the *PSNR* value of BM3D improved by combining feature maps of two channels in any two layers using an exhaustive method. By comparing the denoising effects of various combinations under these two layers, the *PSNR* value of the combination with the best denoising effect is selected as the optimal *PSNR* value for these two layers. Table 2 shows the optimal *PSNR* values for any two layers.

From Table 2, it can be seen that the largest terms of *PSNR* appear in the combination of layers 4&4. In addition, the denoising effect of the combination containing feature layer 2 is not good, especially the denoising effect of the 2&2 combination is the worst in each image. Further observation reveals that the *PSNR* values of various combinations exhibit a pattern of 1&2 < 1&3 < 1&4, 2&2 < 2&3 < 2&4, indicating that the denoising effect of the feature map of the later layer is better than that of the front layer. This is because the structure of the UNet denoising network is similar to the Autoencoder, and feature layer 2 is located in the down-compression process, so the feature map of this layer will become fuzzy, and the features are not obvious. Therefore, the effect of block-matching with the matching of feature layer 2 is not good. Feature layer 3 is located in the up-decoding process. After denoising by the network, the feature map of this layer begins to become clear, and the features are obvious. Feature layer 4 is located in the last layer of the last process, which is the output of the UNet network. Therefore, the feature map image of this layer is the clearest and the features are also the most obvious. Based on the above experimental results and analysis, the improved BM3D algorithm in the subsequent experiments in this paper adopts a combination of feature maps of layers 4&4.

### 4.3. Performance Comparison of Different Denoising Methods

To evaluate the performance of the improved BM3D algorithm proposed in this paper, we compared two classic nonlocal mean denoising algorithms, BM3D and NLM, respectively. We also selected three advanced deep-learning denoising networks, namely BM3D-Net [16], DIVA [17] and UNet. In addition, a comparison was made with a sparse encoding-based denoising algorithm NCSR [37], and the experimental results are shown in Table 3.

**Figure 6:**
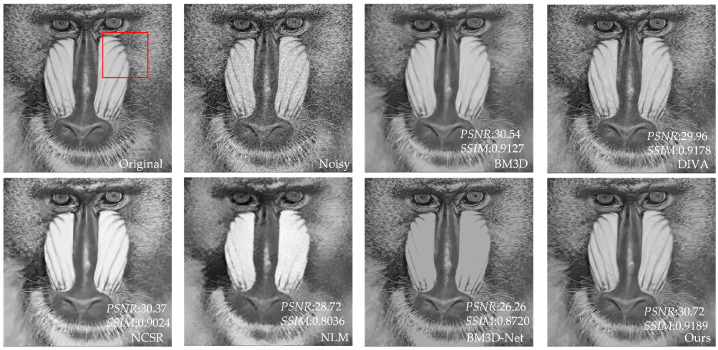
The denoising results of Baboon with σ=10 under six denoising algorithms (the red box represents the local details to be displayed in Figure 7, similar to the following).

**Figure 7:**
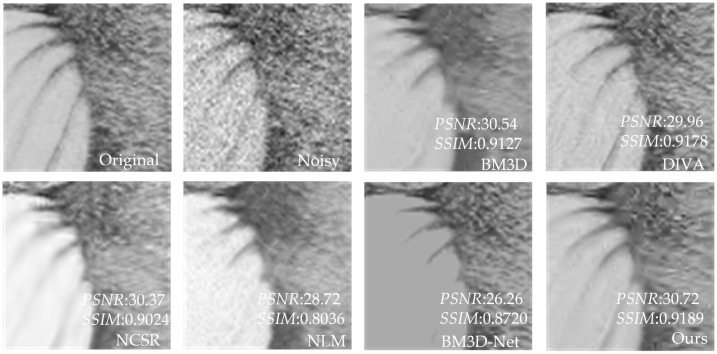
Local denoising results of Baboon with σ=10 under six denoising algorithms.

**Figure 8:**
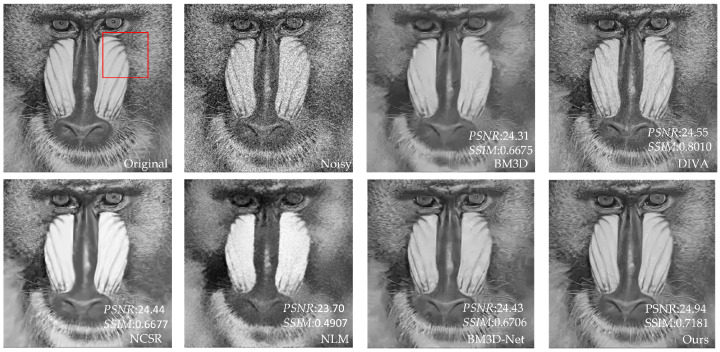
The denoising results of Baboon with σ=30 under six denoising algorithms.

**Figure 9:**
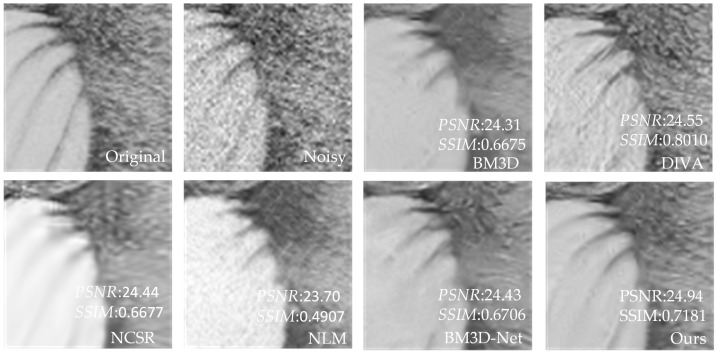
Local denoising results of Baboon with σ=30 under six denoising algorithms.

**Figure 10:**
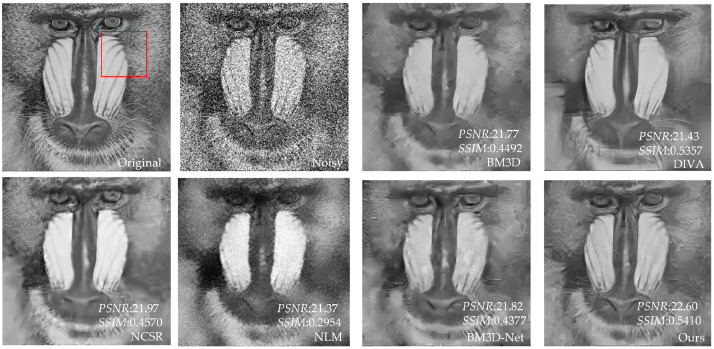
The denoising results of Baboon with σ=50 under six denoising algorithms.

**Figure 11:**
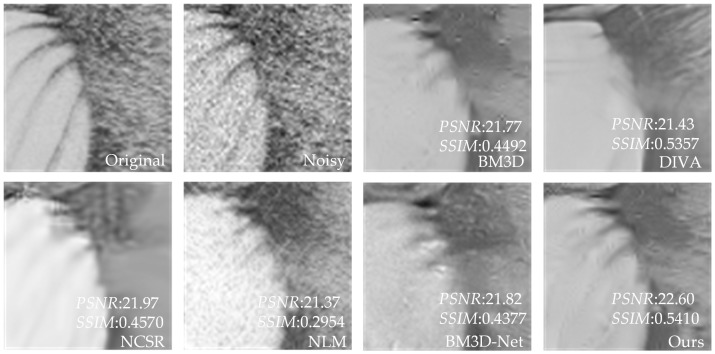
Local denoising results of Baboon with σ=50 under six denoising algorithms.

Table 3 shows that our proposed method has better *PSNR* and *SSIM* values than the other four methods. Compared to UNet, our method has an average increase in *PSNR* of 3.24 dB, and an average increase in *SSIM* of 0.2181 compared to NLM. Compared with BM3D and NCSR, our method has an average increase of 0.26–0.39 dB in *PSNR* value and 0.0076–0.011 in *SSIM* value. Compared with advanced DIVA and BM3D-Net, our method has some advantages in *PSNR*. In addition, for individual images, DIVA’s *SSIM* is better than our method, but overall, our method has better denoising performance. To further demonstrate that our method outperforms the other methods in terms of denoising performance, we utilize the image of Baboon as an example to visualize the denoising effects of various methods with different noise levels. In Figure 6, Figure 7, Figure 8, Figure 9, Figure 10 and Figure 11, “original” represents the original image without noise, “noisy” represents the noisy image, and the rest are the denoised images of BM3D, DIVA, NCSR, NLM, BM3D-Net and Our method in order.

Figure 6, Figure 7, Figure 8, Figure 9 and Figure 10 show the denoising effects of six denoising algorithms on Baboon with noise levels of σ=10, σ=30, and σ=50, respectively. Figure 7, Figure 8, Figure 9, Figure 10 and Figure 11 show the local denoising effects of six denoising algorithms on Baboon with noise levels of σ=10, σ=30, and σ=50, respectively. It can be seen that after denoising with BM3D, NCSR, NLM, and BM3D-Net algorithms, the complex texture areas such as Baboon’s nose bridge, facial hair, and beard become smoother, resulting in a certain degree of distortion. Although the image denoised by the DIVA algorithm retains a certain degree of texture, its denoising effect on noise is not particularly ideal. Our method not only effectively removes noise, but also preserves good texture and has high visual quality in the denoised image. With the increase in noise level, other algorithms’ denoised images exhibit blurry and smooth phenomena. Although our method also exhibits some degree of distortion, it still has a certain visual quality compared to others. The above experiments can verify that the proposed method for block-matching on feature maps fully considers the deep-level features of images at different scales, therefore improving the accuracy of block-matching. Moreover, the proposed method adopts *SSIM* to improve the similarity measurement criterion for block-matching, which further improves the visual quality of the denoised image.

### 4.4. Comparison of Various Algorithms under Different Noise Levels

**Figure 12:**
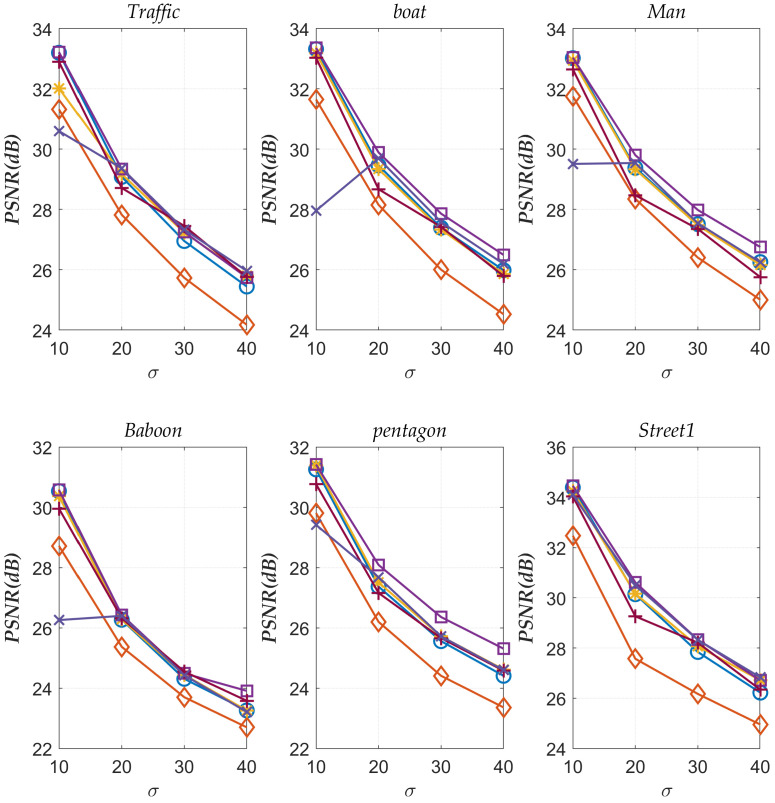
*PSNR* values of various denoising methods under different noise levels. “□”: Improved BM3D; “+”: DIVA; “*”: NCSR; “∘”: BM3D; “⋄”: NLM; “x”: BM3D-Net.

Table 3 presents the numerical results of various denoising algorithms at noise level σ=10. To verify that the proposed method performs better than other methods at different noise levels, six images with noise levels of σ=10, σ=20, σ=30 and σ=40 are selected for the experiment. The obtained *PSNR* values are visualized in Figure 12.

From Figure 12, it can be seen that as the noise level increases, the denoising performance of various denoising methods decreases. This is because the more noise, the less detailed information of the image is presented, making the algorithm unable to utilize more features of the image, resulting in a decrease in denoising performance. Overall, the method proposed in this paper exhibits the best performance under various noise levels and uniformly outperforms other methods. In addition, we can see from the graph that for individual images, the *PSNR* value of the BM3D-Net algorithm at a noise level of σ=10 is lower than the *PSNR* value at a noise level of σ=20. The main reason for this phenomenon is that the network should be trained separately for different noise levels. In addition, this is also related to the data. For example, differences in noise distribution and changes in image content and structure. These characteristics can also lead to abnormal performance of algorithms at certain noise levels.

### 4.5. Ablation Experiment

We conduct ablation experiments to better analyze the contributions of different components of the method proposed in this paper. In the following example, we test four methods with different components.

(1) BM3D: Traditional BM3D method.

(2) BM3D + *SSIM*: A method for improving traditional BM3D based on *SSIM*.

(3) BM3D + Feature: A method for improving traditional BM3D based on UNet de-noising network feature maps.

(4) BM3D + *SSIM* + Feature: A method for improving traditional BM3D based on *SSIM* and UNet denoising network feature maps.

Based on the experimental results in Table 4, the following observations can be obtained. First, the denoising effect of the improved BM3D based on *SSIM* is better than that of the traditional BM3D method, which is reflected in the average increase of 0.06 dB in *PSNR* value and 0.0019 in *SSIM* value. This is because the *SSIM* considers the structure, brightness, and contrast information of the image, so the denoised image has higher visual quality. Second, the denoising effect of the improved BM3D based on UNet denoising network feature maps is superior to the traditional BM3D method, specifically reflected in an average increase of 0.19 dB in *PSNR* value and 0.0055 in *SSIM* value. This is because block-matching on feature maps takes into account the depth features of different scales of the noisy image, resulting in more accurate block-matching results and better denoising effects. Third, the denoising effect of the improved BM3D based on *SSIM* and UNet denoising network feature maps is higher than the other three methods. Compared with the traditional BM3D algorithm, the average *PSNR* value increases by 0.26 dB, and the average *SSIM* value increases by 0.0076. The above experimental results verify that both the UNet denoising network feature map and the improved BM3D algorithm based on *SSIM* proposed in this paper can improve the denoising performance of the traditional BM3D algorithm.

### 4.6. Application Case

**Figure 13:**
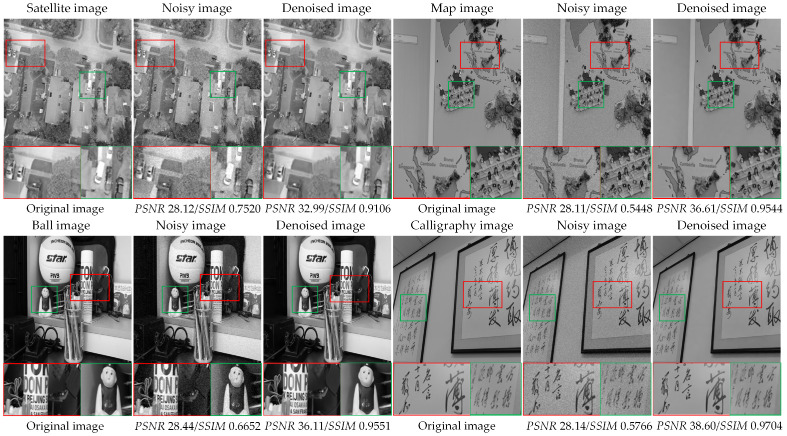
The denoising image results of real-life images (the red and green boxes indicate the local details to be displayed).

To verify the denoising potential of the denoising method proposed in this paper, we selected some real-life image data for denoising, including one satellite image, one map image, one ball image, and one calligraphy image. As shown in Figure 13, the satellite image is from the FloodNet [38] dataset, and the remaining three images are from the PolyU [39] dataset.

We add Gaussian noise with a noise level of σ=10 to each of the four images and then utilize the improved BM3D method proposed in this paper for denoising. In Figure 13, we present the original image, noisy image, and denoised image of each image, and enlarge the local details of each image below the image. In addition, we also list the *PSNR* and *SSIM* numerical results of the noisy and denoised images at the bottom of the image. On the one hand, it can be seen from the denoised image and the enlarged image of local details that the proposed method effectively removes noise from the image and preserves the detailed features of the image clearly. On the other hand, from the numerical results, it can be seen that compared with the *PSNR* and *SSIM* values of noisy images, the proposed method has significantly improved *PSNR* and *SSIM*. From the above analysis, we can conclude that the improved BM3D method proposed in this paper also has excellent denoising effects on real-life images and has good denoising potential.

## 5. Conclusions

We propose an improved BM3D denoising algorithm based on the UNet denoising network feature map and Structural Similarity Index (*SSIM*). Compared to the traditional BM3D algorithm that directly performs block-matching on the noisy image, this method takes into account the deep-level features of different scales of the image and improves the accuracy of block-matching. Moreover, *SSIM* is utilized to improve the similarity measurement criterion, taking into account the structure, brightness, and contrast information of the image, further enhancing the denoising effect of the BM3D algorithm. In addition, it should be pointed out that the time complexity of the method proposed is relatively high, mainly due to the use of the *SSIM* to improve the similarity measurement criterion for BM3D block-matching, which involves *SSIM* calculation between many blocks of the image. In areas with high requirements for image quality, the proposed method can play a good role in non-real-time applications such as image restoration and image enhancement. Finally, through several experiments, the following conclusions can be drawn:

(1) Feature map combinations from different layers of the UNet denoising network are utilized to improve the block-matching process of BM3D, the denoising effect of feature map combinations containing feature layer 2 is poor, while the denoising effect of feature map combinations containing feature layer 4 is excellent.

(2) Compared to the six classic denoising algorithms of traditional BM3D, NLM, UNet, NCSR, DIVA, and BM3D-Net, the improved BM3D method proposed in this paper both has advantages in *PSNR* and *SSIM*. In addition, the proposed method outperforms other methods in denoising images with different noise levels.

(3) The block-matching process of the improved BM3D algorithm proposed in this paper fully considers the deep-level features of the image at different scales, as well as the structure, brightness, and contrast information of the image. After denoising, the detailed features of the image can be well preserved.

(4) The UNet denoising network feature map and *SSIM* in the proposed method in this paper both have a promoting effect on improving the denoising performance of the traditional BM3D algorithm. Using both to improve the denoising effect of the traditional BM3D algorithm is better than using either of them alone.

## Figures and Tables

**Table 1:** BM3D algorithm parameter settings.

$N_{1}^{\mathrm{ht}}$	$N_{2}^{\mathrm{ht}}$	$p^{\mathrm{ht}}$	$\tau_{1}^{\mathrm{ht}}$	$\lambda_{\mathrm{thr}2D}$	$\lambda_{\mathrm{thr}3D}$	$\lambda_{\mathrm{thr}}$	$N_{1}^{\mathrm{wie}}$	$N_{2}^{\mathrm{wie}}$	$p^{\mathrm{wie}}$	$\tau_{1}^{\mathrm{wie}}$
8	8	1	325	0.82	0.75	2.7	8	16	3	400

**Table 2:** Denoising results of improved BM3D algorithm under different UNet denoising network feature map combinations based on *PSNR*.

Image	Original	1&1	1&2	1&3	1&4	2&2	2&3	2&4	3&3	3&4	4&4
Cameraman	34.39	34.37	34.26	34.28	34.37	33.66	33.89	33.95	34.28	34.40	**34.59**
Man	33.02	32.98	32.88	32.95	32.98	32.42	32.63	32.71	32.92	33.02	**33.24**
boat	33.32	33.22	33.15	33.28	33.28	32.69	32.89	32.97	33.30	33.36	**33.58**
Baboon	30.54	30.54	30.53	30.55	30.56	30.35	30.47	30.50	30.54	30.56	**30.68**
Flowers1	31.96	32.14	32.09	32.12	32.14	31.89	31.97	32.00	32.14	32.14	**32.25**
Computer	33.51	33.34	33.03	33.26	33.35	32.26	32.61	32.77	33.21	33.46	**33.75**
Traffic	33.20	33.14	33.03	33.05	33.13	32.57	32.76	32.84	33.09	33.21	**33.30**
Alley	32.42	32.36	31.94	32.36	32.41	31.92	32.13	32.20	32.35	32.47	**32.51**
Trees	29.60	29.62	29.60	29.60	29.61	29.51	29.54	29.55	29.58	29.64	**29.68**
Gardens	30.56	30.60	30.53	30.57	30.64	30.37	30.44	30.47	30.64	30.65	**30.70**
Plaza	34.14	34.20	34.11	34.18	34.33	33.92	34.01	34.12	34.34	34.38	**34.41**
pentagon	31.26	31.23	31.04	31.18	31.24	30.63	30.82	30.91	31.14	31.34	**31.47**
Street1	34.39	34.35	34.21	34.21	34.34	33.70	33.95	34.02	34.28	34.41	**34.61**
Building2	33.71	32.99	33.00	33.27	33.71	33.21	33.31	33.39	33.60	33.70	**33.72**
mean	32.57	32.51	32.39	32.49	32.58	32.08	32.24	32.31	32.53	32.62	**32.75**

The image noise level used in this experiment is σ=10, with bold values representing the maximum value of the row. The first column “original” represents the traditional BM3D algorithm, and the last row “mean” represents the average of each column.

**Table 3:** Denoising results of different algorithms based on *PSNR* and *SSIM* (σ=10).

Image	BM3D	UNet	NLM	DIVA	BM3D-Net	NCSR	OURS
Cameraman	34.39/0.9285	30.38/0.8492	32.26/0.5279	33.89/0.9240	29.18/0.9189	34.19/0.9312	**34.64/0.9338**
Man	33.02/0.9132	30.91/0.8641	31.75/0.7438	32.65/0.9013	29.51/0.8947	32.94/0.9097	**33.29/0.9208**
boat	33.32/0.9207	31.43/0.8827	31.65/0.7248	33.03/**0.9369**	27.96/0.8905	33.19/0.9159	**33.63**/0.9295
Computer	33.51/0.9422	30.92/0.9040	31.55/0.7333	33.18/0.9373	25.66/0.9034	33.27/0.9398	**33.79/0.9490**
Traffic	33.20/0.9304	30.58/0.8835	31.32/0.6941	32.90/0.9295	30.60/0.9305	33.02/0.9320	**33.36/0.9335**
Baboon	30.54/0.9127	27.89/0.8468	28.72/0.8036	29.96/0.9178	26.26/0.8720	30.37/0.9024	**30.72/0.9189**
Flowers1	31.96/0.8746	29.56/0.8041	30.03/0.5810	31.76/**0.9356**	31.64/0.8863	32.01/0.8792	**32.34**/0.8905
Gardens	30.56/0.9209	26.47/0.8417	27.41/0.7592	30.04/0.9118	19.92/0.8744	30.34/0.9119	**30.76/0.9291**
Trees	29.60/0.9361	25.18/0.8363	26.62/0.8470	28.91/**0.9489**	21.78/0.8395	29.32/0.9267	**29.72**/0.9397
pentagon	31.26/0.9040	27.76/0.7878	29.81/0.7800	30.77/0.9044	29.42/0.8754	31.39/0.8968	**31.67/0.9157**
Street1	34.39/0.9312	31.92/0.8952	32.47/0.6045	34.04/0.9243	34.10/0.9325	34.25/0.9319	**34.70/0.9376**
mean	32.34/0.9195	29.36/0.8541	30.33/0.7090	31.92/0.9247	27.82/0.8926	32.21/0.9161	**32.60/0.9271**

The numerical results in the table are A/B, where A represents *PSNR*, and B represents *SSIM*. The larger the values of A and B, the better the denoising performance of the algorithm. The last row “mean” represents the average of each column, and the bold values represent the maximum value of that row.

**Table 4:** Denoising results of different components of the method proposed in this paper based on *PSNR* and *SSIM* (σ=10).

Image	BM3D	BM3D + *SSIM*	BM3D + Feature	BM3D + Feature + *SSIM*
Cameraman	34.39/0.9285	34.42/0.9290	34.59/0.9323	**34.64/0.9338**
Man	33.02/0.9132	33.06/0.9151	33.24/0.9194	**33.29/0.9208**
boat	33.32/0.9207	33.37/0.9230	33.58/0.9277	**33.63/0.9295**
Computer	33.51/0.9422	33.56/0.9430	33.75/0.9481	**33.79/0.9490**
Traffic	33.20/0.9304	33.25/0.9314	33.30/0.9317	**33.36/0.9335**
Baboon	30.54/0.9127	30.59/0.9145	30.68/0.9176	**30.72/0.9189**
Flowers1	31.96/0.8746	32.06/0.8784	32.25/0.8871	**32.34/0.8905**
Gardens	30.56/0.9209	30.62/0.9229	30.70/0.9269	**30.76/0.9291**
Trees	29.60/0.9361	29.63/0.9372	29.68/0.9384	**29.72/0.9397**
pentagon	31.26/0.9040	31.37/0.9085	31.47/0.9100	**31.67/0.9157**
Street1	34.39/0.9312	34.46/0.9325	34.61/0.9359	**34.70/0.9376**
mean	32.34/0.9195	32.40/0.9214	32.53/0.9250	**32.60/0.9271**

The numerical results in the table are A/B, where A represents *PSNR*, and B represents *SSIM*. The larger the values of A and B, the better the denoising performance of the algorithm. The last row “mean” represents the average of each column, and the bold values represent the maximum value of that row.

## Data Availability

Not applicable.
